# Acute toxicity of 31 different nanoparticles to zebrafish (*Danio rerio*) tested in adulthood and in early life stages – comparative study

**DOI:** 10.2478/intox-2013-0012

**Published:** 2013-06

**Authors:** Jevgenij A. Kovrižnych, Ružena Sotníková, Dagmar Zeljenková, Eva Rollerová, Elena Szabová, Soňa Wimmerová

**Affiliations:** 1 Slovak Medical University in Bratislava, Limbová 12, 833 03, Bratislava, Slovakia; 2 Institute of Experimental Pharmacology and Toxicology, Slovak Academy of Sciences, Dúbravská 9, 841 04 Bratislava, Slovakia

**Keywords:** zebrafish, fish eggs, acute toxicity, teratogenicity, nanoparticles

## Abstract

At present, nanoparticles are beginning to influence our lives in many ways and understanding the environmental health and safety aspect of nanomaterials has become a crucial issue. The aim of the work was to assess and compare the acute toxicity of 31 different nanomaterials to fish mature individuals *Danio rerio* with that to fish early life stages on using evaluation of the 48- and 96- hour LC_50_ values. A further aim was to evaluate teratogenicity of the nanoparticles tested to fish eggs. The nanoparticles tested were: 8 pure metals, 10 metal oxides, 5 other metal compounds and their mixtures, 2 silicon compounds, 3 calcium compounds, and 3 carbon compounds. Using 48-h and 96-h tests of acute toxicity (according to OECD 203), we evaluated mortality data, LC_50_ values, occurrence of malformations, as well as hatching time. In our study, 6 kinds of nanoparticles – calcium oxide, copper, copper in the form of oxide and CuZnFe_4_O_4_, magnesium oxide, and nickel – caused cumulative mortality. Two kinds of nanoparticles – copper and silver – were toxic for fish with LC_50_ values of approximately 3 mg/L. We did not observe marked differences between the 48-hour and 96-hour acute toxicity LC_50_ values, yet the possibility to evaluate hatching time in the 96-h acute fish toxicity test seems to be an advantage against that of the 48-hour toxicity.

## Introduction

The environmental hazard to vertebrates in aquatic systems is evaluated by performing acute fish experiments (Fish, Acute toxicity test – OECD 203, 1992), the most frequent method for acute toxicity identification in Europe. At present, the acute toxicity of chemicals to fish is most commonly estimated by means of a short-term test, predominantly on juvenile or adult zebrafish. Due to the implementation of the Three Rs (Reduction, Refinement and Replacement) in animal welfare over the last few years, the number of animals used in acute toxicity tests has been reduced, yet it still remains high. With the introduction of the new European Registration, Evaluation, Authorisation and Restriction of Chemicals (REACH) system, this number is likely to increase dramatically. The OECD 203 regulation, although the most sparing, requires 42 sexual mature fish individuals for the main test. Laboratories dealing with evaluation of the toxicity of chemicals and their mixtures need thousands of fish individuals. One of the promising alternative approaches to classical acute fish toxicity testing with live fish is the Fish Embryo Toxicity (OECD, 2006). Major advantages of zebrafish embryos are that they are readily permeable to small molecules added to their incubation medium and the transparent chorion enables an easy observation of development (Zon & Peterson, 2005; Wheeler & Brandli, 2009). Assays of acute toxicity (LC_50_ estimation) in embryos can also include screening for developmental disorders as an indicator of teratogenic effects. The test is utilized for toxicity determination of huge amounts of different chemicals, among them also nanoparticles.

In nanotechnology, nanoparticles are particles that have one dimension between 1 nm and 100 nm in size. Manufactured nanomaterials possess enhanced or even unique physicochemical properties, such as nanoscale size effects, increased surface area, as well as unique electric, thermal, mechanical, and imaging properties (Colvin, 2003). The properties of many conventional materials change when formed from nanoparticles. This is mainly the result of a greater surface area per weight of nanoparticles compared to larger particles; this property causes them to be more reactive with other molecules. Further, the toxicity of nanoparticles can be changed in contrast to a bulk chemical.

At present, debates are going on concerning the advantages of fish acute toxicity testing on fish embryos over that on adult fish individuals. Our work is a contribution to this debate. The work is comparing acute toxicity of 31 different nanomaterials on fish mature individuals *Danio rerio* with that on fish early life stages by using evaluation of the 48- and 96-hour LC_50_ values. A further aim was to evaluate teratogenicity of the nanoparticles tested on fish eggs.

## Methods

The following nanomaterials were tested: 8 pure metals, 10 metal oxides, 5 other metal compounds and their mixtures, 2 silicon compounds, 3 calcium compounds, and 3 carbon compounds from Sigma-Aldrich Chemie GmbH, Germany (Tables 1 and 2). The required concentrations of nanomaterials were prepared by two ways. Nanomaterials which encroach a small volume: stock dispersion of the required amount of chemical was prepared in 100 ml tap water using an ultrasound homogenizer Sonopuls HD 2070 and final concentrations were prepared by dilution of the stock dispersion with tap water. Nanomaterials which encroach a big volume: each concentration tested was prepared separately by homogenization of the required amount of chemical with 100 ml tap water and consecutively by its dilution in the required volume of tap water. The time of preparation of dispersion depended on the kind of material and ranged from 10 minutes to 4 hours

**Table 1:** Toxicity parameters of nanoparticles of pure metals and metal compounds tested in adult fish and eggs of zebrafish *Danio rerio* and teratogenicity of these nanoparticles.

Nanoparticle	Stage	n	48-h LC_50_ mg/L	96-h LC_0_mg/L	96-h LC_50_mg/L	96-h LC_100_mg/L	Teratogenicity
Aluminum oxide(Al_2_O_3_) <50 nm	adult	20	>800.0	ND	>800.0	ND	
	eggs	20	>800.0	ND	>800.0	ND	PH (*p=*0.033)
Aluminum titanate(Al_2_O_3_.TiO_2_) <25 nm	adult	20	>1600.0	ND	>1600.0	ND	
	eggs	20	>1600.0	ND	>1600.0	ND	no
Barium ferrite(BaFe_12_O_19_) <100 nm	adult	20	>1600.0	ND	>1600.0	ND	
	eggs	20	>1600.0	ND	>1600.0	ND	PH (*p=*0.087)
Bismuth oxide(Bi_2_O_3_) <100 nm	adult	20	>1600.0	ND	>1600.0	ND	
	eggs	20	>1600.0	ND	>1600.0	ND	no
Chromium oxide(Cr_2_O_3_)	adult	20	>1600.0	ND	>1600.0	ND	
	eggs	20	>1600.0	ND	>1600.0	ND	no
CopperCu <100 nm	adult	30	4.2	1.0	3.8	10.0	
	eggs	40	24.0	5.0	24.0	ND	DH, M
Copper oxide(CuO) <50nm	adult	90	400.0	12.5	400.0	ND	
	eggs	30	960.0	400.0	840.0	1600.0	no
Copper zinc iron oxide(CuZnFe_4_O_4_) <100 nm	adult	50	390.0	100.0	320.0	ND	
	eggs	30	ND	800.0	2600.0	ND	M?
Gold(Au) <100 nm	adult	20	>200.0	ND	>200.0	ND	
	eggs	20	>200.0	ND	>200.0	ND	no
Iron nickel oxide(NiFe_2_O_4_) <50 nm	adult	20	>1600.0	ND	>1600.0	ND	
	eggs	20	>1600.0	ND	>1600.0	ND	PH (*p=*0.011)
Iron oxide(Fe_2_O_3_) <50 nm	adult	20	>1600.0	ND	>1600.0	ND	
	eggs	20	>1600.0	ND	>1600.0	ND	PH (*p=*0.033)
Magnesium hydroxide(Mg(OH)_2_) <100 nm	adult	20	>1600.0	ND	>1600.0	ND	
	eggs	20	>1600.0	ND	>1600.0	ND	no
Magnesium oxide(MgO) <50 nm	adult	30	140.0	ND	140.0	400.0	
	eggs	20	>3200.0	1600.0	>3200.0	ND	no
Nickel(Ni) <100 nm	adult	20	>400.0	ND	>400.0	ND	
	eggs	20	>400.0	ND	>400.0	ND	no
Nickel oxide(NiO)	adult	30	760.0	200.0	420.0	800.0	
	eggs	30	1700.0	800.0	1300.0	ND	DH, M
Palladium(Pd) <25 nm	adult	20	>100.0	ND	>100.0	ND	
	eggs	20	>100.0	ND	>100.0	ND	PH (*p=*0.020)
Platinum(Pt) <50 nm	adult	20	>100.0	ND	>100.0	ND	
	eggs	20	>100.0	ND	>100.0	ND	no
Silver(Ag)	adult	30	2.9	1.3	2.9	10.0	
	eggs	40	2.7	2.0	2.7	10.0	M
Tin(Sn) <150 nm	adult	20	>800.0	ND	>800.0	ND	
	eggs	20	>800.0	800.0	>800.0	ND	no
Tin oxide(SnO_2_) <100 nm	adult	20	>1600.0	ND	>1600.0	ND	
	eggs	20	>1600.0	ND	>1600.0	ND	PH (*p=*0.081)
Titanium oxide(TiO_2_) <100 nm	adult	20	>1600.0	ND	>1600.0	ND	
	eggs	20	>1600.0	ND	>1600.0	ND	PH (*p=*0.020)
Tungsten(W) <150 nm	adult	20	>1600.0	ND	>1600.0	ND	
	eggs	20	>1600.0	ND	>1600.0	ND	PH (*p=*0.087)
Tungsten oxide(WO_3_) <100 nm	adult	20	>1600.0	ND	>1600.0	ND	
	eggs	20	>1600.0	ND	>1600.0	ND	no

LC_100_ - minimum concentration causing 100% mortality; LC_0_ - maximum concentration causing no mortality; ND - not determined; n - number of fish used for calculation of LC_50_ value; *p* - statistical significance; PH - premature hatching; DH - delayed hatching; M - malformations; no - concentration ≤100 mg/L causing no teratogenicity

**Table 2:** Toxicity parameters of other nanoparticles tested in adult fish and eggs of zebrafish *Danio rerio* and teratogenicity of these nanoparticles.

Nanoparticle	Stage	n	48-h LC_50_mg/L	96-h LC_0_mg/L	96-h LC_50_mg/L	96-h LC_100_ mg/L	Teratogenicity
Calcium phosphateamorphous <100 nm	adult	20	>1600.0	ND	>1600.0	ND	
	eggs	20	>1600.0	ND	>1600.0	ND	no
Calcium oxide(CaO) <160 nm	adult	30	260.0	100.0	260.0	400.0	
	eggs	30	290.0	200.0	290.0	400.0	no
Carbon(C) <50 nm	adult	20	>1600.0	ND	>1600.0	ND	
	eggs	20	>1600.0	ND	>1600.0	ND	no
Carbon nanotube7-15×3–6×0.5–200 nm	adult	20	>200.0	ND	>200.0	ND	
	eggs	20	>200.0	ND	>200.0	ND	no
Fullerene(C60)	adult	20	>200.0	ND	>200.0	ND	
	eggs	20	>200.0	ND	>200.0	ND	no
Hydroxyapatite (Ca_5_HO_13_P_3_) <200 nm	adult	20	>1600.0	ND	>1600.0	ND	
	eggs	20	>1600.0	ND	>1600.0	ND	no
Silicon nitride(Si_3_N_4_) <50 nm	adult	20	>1600.0	ND	>1600.0	ND	
	eggs	20	>1600.0	ND	>1600.0	ND	no
Silicon dioxide(SiO_2_)	adult	20	>1600.0	ND	>1600.0	ND	
	eggs	20	>1600.0	ND	>1600.0	ND	no

LC_100_ - minimum concentration causing 100% mortality; LC_0_ - maximum concentration causing no mortality; ND - not determined; n - number of fish used for calculation of LC_50_ value; no – concentration ≤100 mg/L causing no teratogenicity

### Acute toxicity

Animal experiments were performed in a laboratory fulfilling the criteria of Good Laboratory Practice. They were conducted in accordance with the EEC Directive of 1986; 86/609/EEC and approved by the State Veterinary and Food Administration of the Slovak Republic. Tests on zebrafish eggs were performed according to OECD 203 (1992) and according to the OECD Guideline for Testing of Chemicals 210, Fish, Early Life Stage Toxicity Test (OECD 210, 1992). Fertilized eggs of zebrafish were sorted in the 12^th^ stage (very late blastula) (Hisaoka and Battle, 1958), corresponding to 2.5–3 h after fertilization (Kimmel *et al.*,^1995^). Only eggs of the same quality were used in the experiments. No spontaneous defects of development occurred in embryos after exceeding this stage of development and survival of embryos in control conditions was 100%.

The exposure of eggs to different chemicals was carried out for four days in covered 20 mL Petri dishes with occasional stirring of their content. First, preliminary tests were performed to determine a value close to LC_50_ for each selected chemical. Then, 5 concentrations (below and above the LC_50_ value) were selected using a spacing factor in the range of 2.0 in dependence on the toxicity of an individual chemical to determine the exact LC_50_. The number of eggs in each experimental and control group was 10. Fish embryonic development was observed directly in Petri dishes using a binocular microscope Olympus SZ 1145 TR (Japan).

The exposure of sexually mature individuals of zebrafish to the solution with or without the nanoparticles tested was performed in covered 5 L glass tanks according to OECD 203 (1992). The number of fish in each experimental and control group was 10. The ratio fish/water was 1 g of fish weight to 1.8–2.0 L of water. All toxicity tests were static. Control groups were kept in 25±1 °C water without chemicals, total hardness of water was dgH = 13 N°, the light-dark regimen was 12 h light/12 h darkness, oxygen concentration >60%, pH = 8.3–8.6. The pH values were increased in CaO solutions (in 100 mg/L - pH 10.5, in 400 mg/L - pH 11.8, in 800 mg/L - pH 12.4), in MgOH solutions (in the concentration 1600 mg/L - pH 10.2), and in MgO solutions (from 100 to 800 mg/L - pH 10.4 to 11.1). In the other solutions, pH values matched with control solutions.

The LC_50_ values of the 48- and 96-h exposure were calculated according to the Lichtfield-Wilcoxon method (Lichtfield & Wilcoxon, 1949).

### Teratogenicity

In addition to acute toxicity tests, we determined also the following teratogenicity criteria: incidence and extent of morphological abnormalities, hatching time and the number of hatched fish. Results were statistically analyzed by the Fisher Exact Test.

## Results

**Figure 1:**
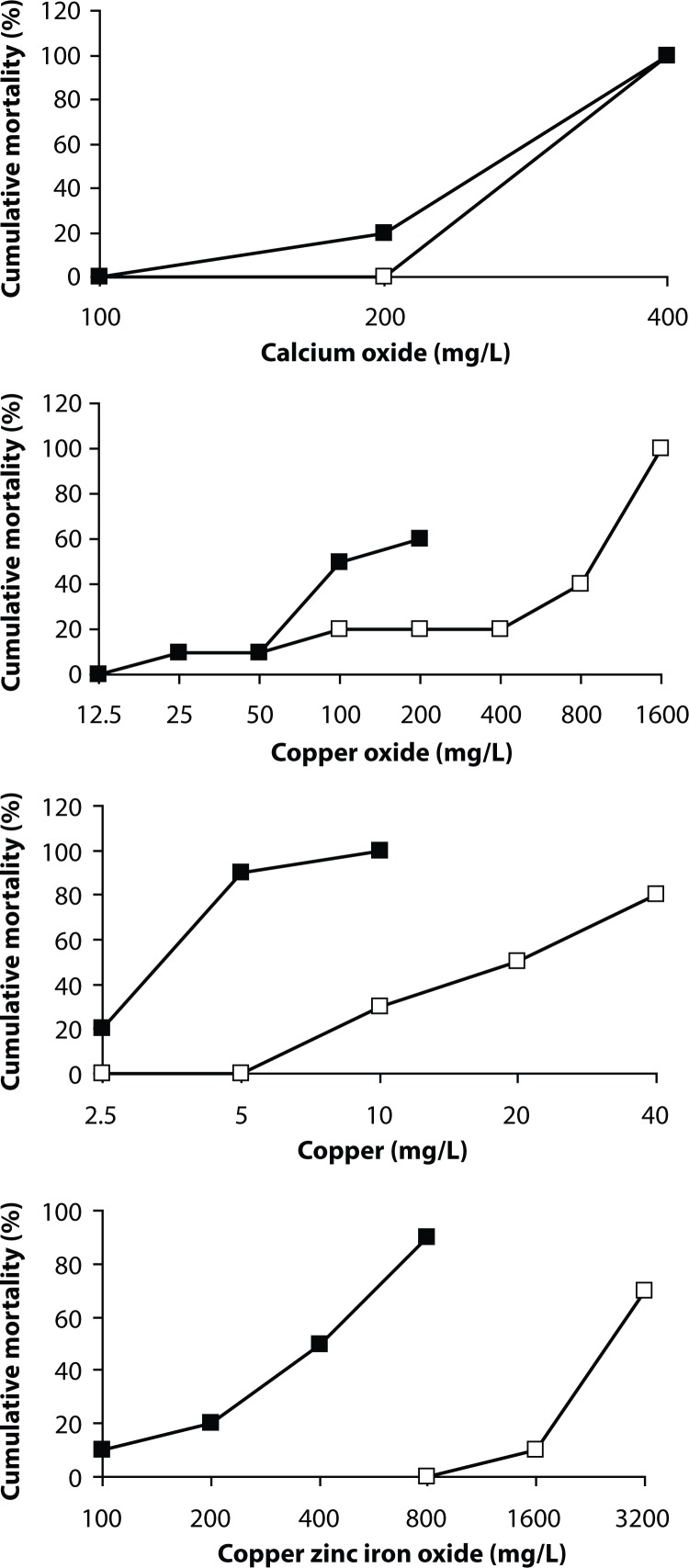
Cumulative mortality of adult zebrafisch *Danio rerio* (■) and fish eggs (□) after 96-hour exposure to four nanoparticles: calcium oxide, copper, copper oxide, copper zinc iron oxide.

**Figure 2:**
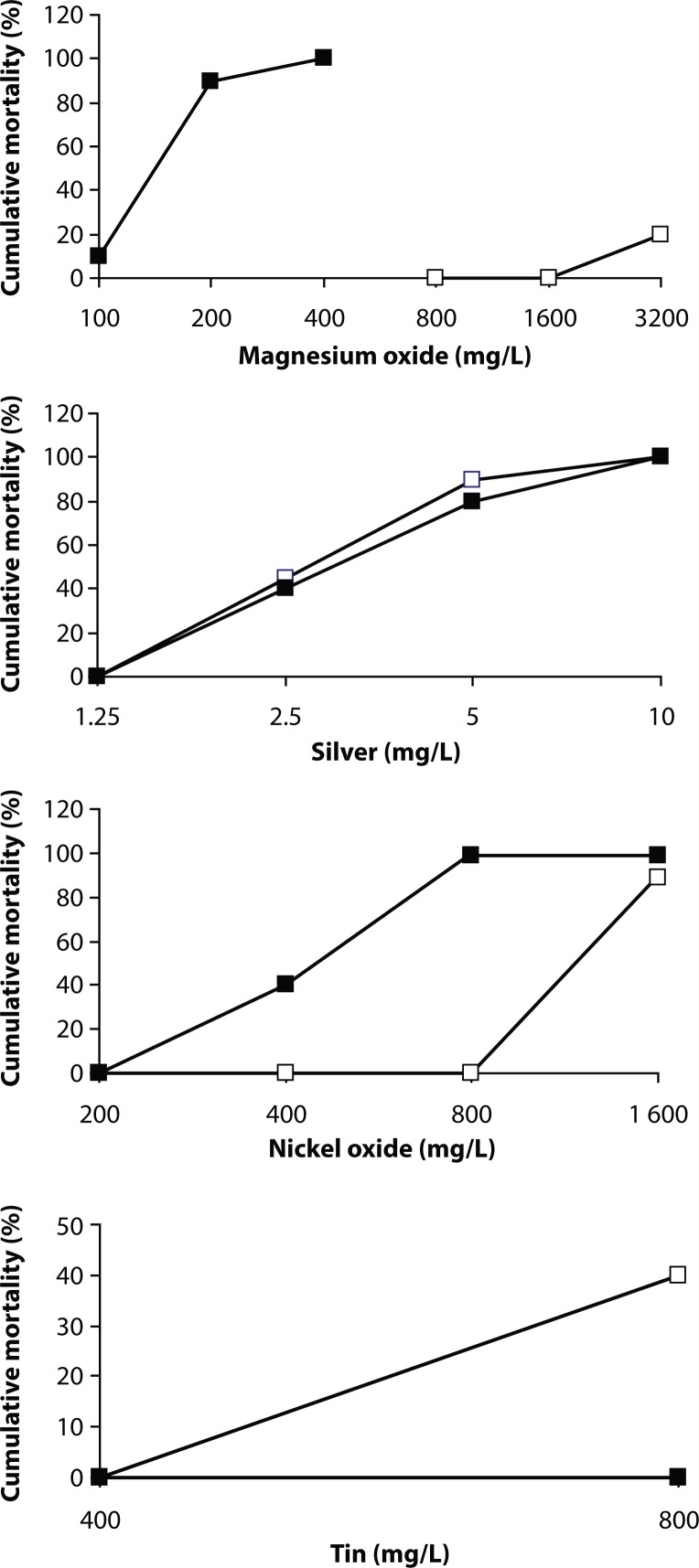
Cumulative mortality of adult zebrafisch *Danio rerio* (■) and fish eggs (□) after 96-hour exposure to four nanoparticles: magnesium oxide, nickel oxide, silver and tin.

Except 8 nanoparticles presented in Figures 1 and 2, cumulative mortality of adult individuals and eggs of zebrafish was not observed. Tables 1 and 2 summarize the LC_50_ values of the 96 h acute toxicity test, the minimum concentration causing 100% mortality within 96 h, and the maximum concentration causing no mortality within 96 h of the same chemical, as well as LC_50_ values of the 48 h acute toxicity test for adult fish and fish eggs and teratogenicity data. From all nanoparticles studied, only silver and copper were found to be toxic according to OECD 203.

In the case of calcium oxide, magnesium oxide and silver nanoparticles, mortality of either fish eggs or adult fish did not increase after 48 hours. The LC_50_ values of the 48-h and 96-h toxicities do not differ (Tables 1 and 2). The adult fish toxicity of copper nanoparticles, however, depended on the time of the presence of the chemical in the incubation medium – the 48-h LC_50_ was 4.2 mg/L, while the 96-h LC_50_ was already 3.8 mg/L. The same situation was in the case of copper oxide – LC_50_ values changed from 960 mg/L to 840 mg/L. As copper zinc iron oxide nanoparticles covered fish eggs so intensively that the embryos were not visible, we preferred to perform only the 96-h toxicity, *i.e.* the time when we could poke out the embryos from the egg cover. A pronounced augmentation of fish mortality was observed by nickel oxide nanoparticles. The 96-h LC_50_ augmented from 760 mg/L to 420 mg/L in adult fish and from 1700 mg/L to 1300 mg/L in fish eggs.

After 96-h incubation of fish eggs with nanoparticles, we evaluated their teratogenicity (Table 3). Copper nanoparticles in all concentrations tested (2.5 to 40 mg/L) induced deformation and almost twofold extent of yolk volume in 100% of embryos. Moreover, higher concentrations caused body deformations in 30–100% embryos: shortening of the tail stem length, its deformation up to breaking. Besides, copper nanoparticles prevented hatching of fish even in the concentrations not inducing mortality (2.5 mg/L). For example, after 96 hours of testing, 70% of embryos were born in control conditions, 10% in the copper concentration of 2.5 mg/L (no mortality), and no embryos were born in the concentration of 5 mg/L (no mortality) (Tables 1 and 3).

**Table 3:** Teratogenicity parameters of nanoparticles of pure metals and metal compounds tested in eggs of zebrafish *Danio rerio.*

Nanoparticle	Concentration mg/L	Yolk deformation %	Body shortening of the embryos %	0ther malformations %	Hatching after 96h (% in control / % in test)
Aluminum oxide (Al_2_O_3_) <50 nm	800.0	0.0	0.0	0.0	0.0/80.0
Barium ferrite (BaFe_12_O_19_) <100 nm	1600.0	0.0	0.0	0.0	0.0/60.0
Copper Cu <100 nm	2.5	100.0	30.0	30.0	70.0/10.0
	5.0	100.0	30.0	30.0	70.0/0.0
	10.0	100.0	57.1	57.1	70.0/0.0
	20.0	100.0	75.0	75.0	70.0/0.0
	40.0	100.0	100.0	100.0	70.0/0.0
Copper zinc iron oxide (CuZnFe_4_O_4_) <100 nm	3200.0	0.0	10.0	10.0	0.0/0.0
Iron nickel oxide (NiFe_2_O_4_) <50 nm	1600.0	0.0	0.0	0.0	0.0/60.0
Iron oxide (Fe_2_O_3_) <50 nm	1600.0	0.0	0.0	0.0	0.0/100.0
Nickel oxide (NiO)	800.0	10.0	0.0	0.0	50.0/0.0
Palladium (Pd) <25 nm	100.0	0.0	0.0	0.0	10.0/70.0
Silver (Ag)	2.5	100.0	100.0	0.0	10.0/10.0
Tin oxide (SnO_2_) <100 nm	1600.0	0.0	0.0	0.0	30.0/100.0
Titanium oxide (TiO_2_) <100 nm	1600.0	0.0	0.0	0.0	10.0/90.0
Tungsten (W) <150 nm	1600.0	0.0	0.0	0.0	0.0/40.0

In the case of CuZnFe_4_O_4_ nanoparticles, we observed one embryo with a body deformation – a shortened tail stem and enlarged pericardial space – at the concentration of 3200 mg/L (Table 1). In the other 9 embryos, no developmental defects occurred in either concentration tested. CuZnFe_4_O_4_ is thus suspected to be a teratogen, at least in very high concentrations.

Silver nanoparticles were found to be highly toxic for fish embryos. They mostly caused disorders leading to death of embryos already during their development. In the concentration of 2.5 mg/L, silver nanoparticles induced yolk deformation and body shortening of the embryos (Table 3).

Nickel oxide nanoparticles did not induce malformations of fish body, but in the concentrations of 800 and 1600 mg/L a mild extent of the volume and stretching of the yolk pouch was observed. After 96 hours, 50% of embryos were born in the control conditions, 20% in nickel oxide nanoparticles (100 mg/L) and 10% in nickel oxide nanoparticles (200 mg/L). In the concentrations of 400 and 800 mg/L no embryo was born (Table 3).

Some nanomaterials tested caused statistically significant premature hatching (PH) of fish in comparison with controls. Conversely, the copper and nickel oxide nanoparticles induced delayed hatching (DH) (Table 1).

## Discussion

Of the 31 nanoparticles tested, 23 were found to be nontoxic up to the concentration of 100 mg/L for adult and embryo zebrafish. The remaining 8 were calcium oxide, magnesium oxide, copper and copper in both forms of oxide, nickel oxide and silver. Nanoparticles of calcium oxide, magnesium oxide, copper and copper in both forms of oxide, nickel oxide and silver, caused cumulative mortality. According to OECD 203, only copper and silver fullfiled toxicity criteria for both adult fish and embryos. Some of the nanoparticles studied, which were found to be non-toxic, were evaluated by other authors equally as non-toxic. For instance, aluminium, cobalt and titanium dioxide nanoparticles were found to be very little toxic to adult zebrafish (Griffitt *et al.*, 2007; 2008; 2011). In agreement with our results, metal nanoparticles of copper were shown to be acutely toxic in zebrafish with an LC_50_ value of 1–1.5 mg/L (Griffitt, 2007; 2008) and classified at the same toxicity level according to ON 46 6807 (1988).

The silver ion and nanoparticles of silver are toxic to fish (Lima *et al.*, 1982, Morgan & Wood, 2004). The 48-hour and 96-hour lethal concentration (LC_50_) values of nanosilver in our experiments were approx. 3mg/L. There are however different reports on fish toxicity in the literature. While Bilberg *et al.* (2012) found the 48-h LC_50_ value to be 84 µg/ L, Griffitt *et al.* (2008) reported a 48-hour nanosilver LC_50_ value of 7.07 mg/L. In another study on zebrafish, Choi *et al.* (2010) reported the 24-hour LC_50_ to be 250 mg/L for silver nanoparticles. In the above mentioned studies, the authors assessed silver nanoparticles of different sizes and with or without different stabilization agents, which seems to be the reason of the differring acute toxicity.

Regulations dealing with categorization of chemical toxicity, *e.g.* ON 46 6807 (1988), classify chemicals with 10-fold toxicity difference at one toxicity level. Taking this into consideration, our results showed that up to the borderline value of 100 mg/L, the toxicity level for two fish life stages (adult fish and embryos) was the same in all 31 nanoparticles tested. Values higher than 100 mg/L were the same in 30 of 31 nanoparticles. The ratio LC_50_ for eggs/LC_50_ for adult fish of 26 nanoparticles was found to be 0.9–1.1. This ratio for nickel oxide was 3.1, copper 6.3, for copper oxide 6.5, copper zinc iron oxide 8.1. Only magnesium oxide nanoparticles belong to different toxicity levels for eggs and for adult fish – they were 23-times less toxic to eggs than to adult fish. Similar results were found in our previous work (Kovrižnych & Urbančíková, 2001), in which we compared acute toxicity of 8 different chemicals, *e.g.* acetochlor, benzene, *etc.* The same toxicity level was determined in 7 out of 8 compounds, whereby up to the borderline value of 100 mg/L it was the same in all chemicals tested.

Several authors published papers comparing acute toxicity of chemicals in adult fish and eggs. In accordance with our results, more authors referred comparable acute toxicity of chemicals to adult fish and fish eggs. Schulte & Nagel (1994) tested acute toxicity of six phenolic compounds to embryos of zebrafish within the first 48 hours of their development. They found that the values of acute toxicity in embryos were similar to those in adult fish. By using a modified Schutle and Nagel's test, Vaughan & Egmond (2010) tested the acute toxicity of a number of anionic, cationic and non-ionic surfactants to embryos of the zebrafish over 48 hours, as a possible alternative to the standard 96-hour fish acute test. The data showed that the embryos appeared to be as sensitive to cationic and non-ionic surfactants as the adult fish, but possibly more sensitive to anionic surfactants. Further, Knöbel *et al.* (2012) confirmed a very strong correlation of the zebrafish embryo to adult fish acute toxicity of organic industrial chemicals with a wide range of physicochemical properties, toxicities, and modes of toxic action. Thus, the majority of chemicals seem to be comparably toxic to eggs and to adult zebrafish and the zebrafish embryo test can be considered a suitable alternative to the adult fish test (Birge *et al.*,^1979^; Schulte & Nagel, 1994; Kovrižnych & Urbančíková, 2001).

Some of the authors however found different acute toxicity of chemicals to different life stages of fish. For instance, Ensenbach *et al.* (1989) investigated the acute toxicities of phenol, 4-chlorophenol, pentachlorophenol, 4-nitrophenol and methanol in eggs of *Danio rerio*. The endpoint, death, was defined as no heart-beat in embryos (by 48 hours). The authors compared this value with the acute toxicities of the chemicals in adult zebrafish (by 96 hours) and found that embryos were not as sensitive to chemicals as adult zebrafish. Cairns (1965) examined the effects of several chemicals on different life stages of various fish species and found that they did not have the same relative response to different toxic materials. In the case of zinc chloride, potassium cyanide and potassium dichromate, the adult zebrafish appeared to be less tolerant than the zebrafish eggs. On the other hand, the zebrafish eggs showed higher sensitivity to naphthenic acids than did the adult zebrafish or the adult bluegill. On the contrary, Laale (1977) proposed that early life stages were more sensitive to chemicals than adult fish. The susceptibility of embryos to chemicals depends on many factors, concerning especially the yet improperly developed enzymatic system in embryos, different ways of absorption of the chemicals in the organism, or differences in metabolic pathways. These could belong to the reasons for the different sensitivity of the embryonic stages of zebrafish to some substances, as compared with adult individuals (Praskova *et al.*, 2011).

Concerning the time duration, the 48-hour zebrafish embryo test can be considered as a suitable alternative to the adult acute fish test for chemicals (Vaughan & Egmond, 2010). This test on embryos can be carried out more quickly than with adult fish, it uses much less space and media, requires less effort, which results in a reduced cost. However, the 96-h acute fish toxicity test allows to evaluate a possible teratogenicity of chemicals during the entire zebrafish embryo development. After 96-h incubation of fish eggs with the majority of nanoparticles tested, we could observe developmental malformations and alterations in the hatching time. The 96-h exposure of zebrafish eggs to nanoparticles for assessment of developmental toxicity parameters and hatching was used also by other authors (Zhu *et al.*, 2007; Fent *et al.*, 2010). Of the 31 nanoparticles tested in our experiments, nine were found to alter the hatching. Although fullerene did not fall within the latter group, Zhu *et al.* (2007) found it to decrease hatching rate. The disparities may have resulted from different mode of preparation of nanoparticles. On balance then, the 96-hour exposure to nanoparticles showed for the first time that aluminium oxide, barium ferrite, copper, iron nickel oxide, iron oxide, palladium, tin oxide, titanium oxide, and tungsten affected the hatching time of zebrafish.

## Conclusion

By using a battery of 31 nanoparticles we found only little differences between LC_50_ toxicity values in eggs and in adult fish. We consider the 96-h acute fish toxicity to have some advantages against the 48-h acute toxicity test because this time interval enables to assess a possible teratogenicity of chemicals during the entire zebrafish embryo development, along with an important toxicity parameter – the hatching time. These observations seem to justify the choice of the 96 h toxicity test.
